# The heart of the matter: secretory pheochromocytoma presenting as recurrent biventricular heart failure (Takotsubo cardiomyopathy)

**DOI:** 10.1093/omcr/omac066

**Published:** 2022-06-23

**Authors:** Lauren M Turner, Hazel Serraro-Brown, Mairi McLaren, Lau Rachel, Charles Mosse

**Affiliations:** Department of Surgery, Division of Critical Care, Canberra Hospital, Garran, ACT, Australia; Department of Surgery, Division of Critical Care, Canberra Hospital, Garran, ACT, Australia; Department of Surgery, Division of Critical Care, Canberra Hospital, Garran, ACT, Australia; Department of Pathology, Canberra Hospital, Garran, ACT, Australia; Department of Surgery, Division of Critical Care, Canberra Hospital, Garran, ACT, Australia

## Abstract

Takotsubo’s syndrome (TS) is an acute, transient cardiomyopathy occurring secondary to physical or emotional stressors through catecholamine excess. Secretory pheochromocytomas have been previously implicated in cases of TS (PTS), however, often present atypically, are associated with reoccurrence, and have higher rates of complications. We describe the case of a 70-year-old female who presented central chest pain, hypotension and electrocardiogram changes on a background of a 6-month prior episode of resolved Takotsubo’s with unknown cause. After progressing to cardiogenic shock with biventricular failure, computerized tomography coronary aortogram revealed an incidental adrenal mass, later proven to be a secretory pheochromocytoma on biochemistry and subsequent histology. PTS has been associated with recurrence and rarely presents as cardiogenic shock. This case highlights the complexity of TS presentations and complications and the diagnostic delays that may occur in PTS.

## INTRODUCTION

Takotsubo’s syndrome (TS) is an acute cardiomyopathy, which causes transient circumferential left ventricular wall motion abnormality in the context of intense physical or emotional stress, in absence of coronary artery compromise on angiography or plaque rupture [1–3]. The name Takotsubo refers to a Japanese octopus trap, which the apical ballooning of the left ventricle in systole bears resemblance to in TS. TS is considerably more common in older (mean [±SD] 66.8 ± 13.0 years) women (9:1), occurs secondary to a myriad of conditions including intracranial insults (e.g. subarachnoid haemorrhage, head injury, stroke, epilepsy and electroconvulsive therapy), psychiatric conditions (e.g. anxiety and depression) or states of catecholamine excess [3]. Circulating catecholamines and the ‘surge effect’ [4] have been proposed to induce damage through either microvascular dysfunction, which results in myocardial stunning [5] or, through direct toxicity on myocardial cells [6]. Pheochromocytoma—an adrenal tumour which can be secretory—is one such cause of catecholamenia. Symptoms from of pheochromocytoma-induced TS (PTS) are frequently more atypical than non-pheochromocytoma-induced TS, presenting a diagnostic challenge, and with greater risk of complications and recurrence [3, 7, 8]. Furthermore, aside from a recently published meta-analysis [8], existing data are limited, consisting of case reports and series. Here, we present the case of an otherwise well, healthy female with two presentations of Takotsubo’s, escalating in severity, before definitive treatment of her adrenal tumour.

## CASE REPORT

A 70-year-old female presented to her local hospital with nausea, central chest pain, palpitations, vomiting and shortness of breath. She was found to be hypotensive, with a moderate troponin rise (150 ng/l) and S–T elevation on electrocardiogram (leads V1–V4). She was transferred to a private regional hospital coronary care unit (CCU) where cardiac investigations including transthoracic echocardiogram (TTE) and angiogram were found to be normal. She reported no recent increase in stress, and her past medical history was significant for previous hemithyroidectomy (2011, currently euthyroid) and osteoarthritis, with no history of diabetes, hypertension or smoking, and no family history of catecholamine-secreting tumours. Notably, she had presented 6-month earlier to her local hospital with an episode of hypotension, chest pain and TTE findings consisting of an ejection fraction of 30%, hypokinetic left ventricle (LV), poorly contracting right ventricle (RV) with dilatation and mild mitral and tricuspid regurgitation. Consistent with TS, symptoms and TTE findings had resolved within 3 months on repeat TTE with commencement of aspirin 100 mg in the morning, simvastatin 20 mg and rampiril 1.25 mg in the evening.

**Figure 1:**
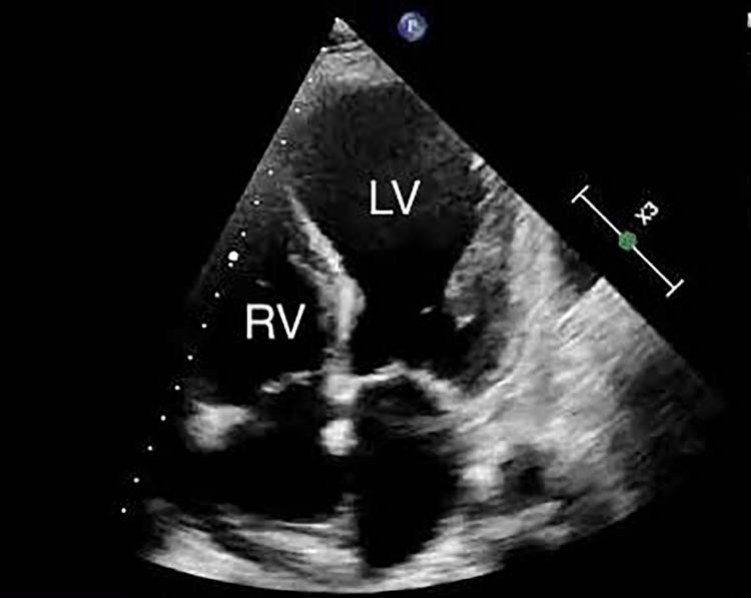
Apical 4 chamber view of left ventricular and right ventricular views shows severe segmental systolic dysfunction. Estimated LVEF was 20% by Simpson’s biplane method.

Twenty-four hours after commencing bisoprolol 12.5 mg BD in CCU, she developed cardiogenic shock with biventricular failure and was transferred to the intensive care unit (ICU) for management. She was profoundly bradycardic (40 bpm), with a troponin of 7327 ng/l, and a left ventricular ejection fraction (LVEF) of ~18–23% with severe regional wall motion abnormalities (severe akinesis in all segments except the base), mildly dilated right ventricle with wall motion abnormality (Fig. 1) and evidence of end organ dysfunction (acute kidney injury and ischaemic hepatitis). She reported no pain however was diaphoretic with ongoing nausea. Milrinone and dobutamine infusions were commenced, resulting in tachycardia up to 200 bpm with atrial fibrillation and rapid ventricular rate (RVR), which was chemically reverted to sinus rhythm with amiodarone, and required no further inotropic support. Computerized tomography (CT) angiogram revealed no significant coronary artery disease and notably her CT aortogram revealed no aortic dissection but an incidental finding of a 5.8 × 4.8 × 5.5 heterogenous mass right-sided adrenal mass.

**Figure 2:**
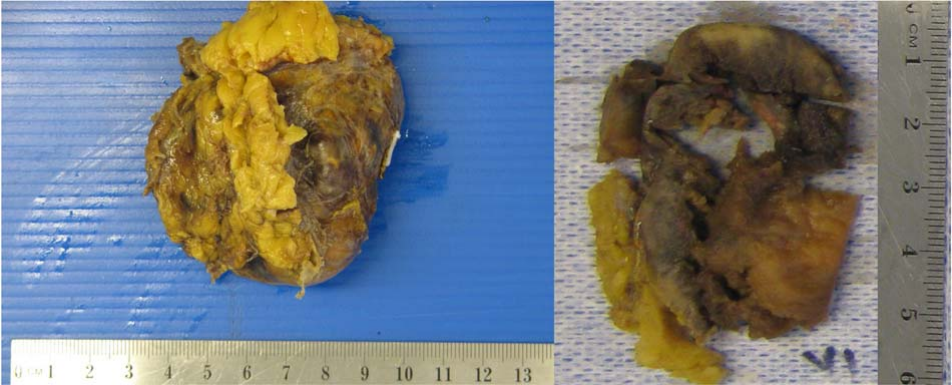
Right adrenalectomy specimen, capsule intact, measuring 60 × 25 × 25 mm, weight 84.1 g. Sectioning revealed a friable, haemorrhagic mahogany brown tumour 35 × 30 × 23 mm, with iatrogenic partial disruption, and unremarkable uninvolved sections of adrenal gland.

**Figure 3:**
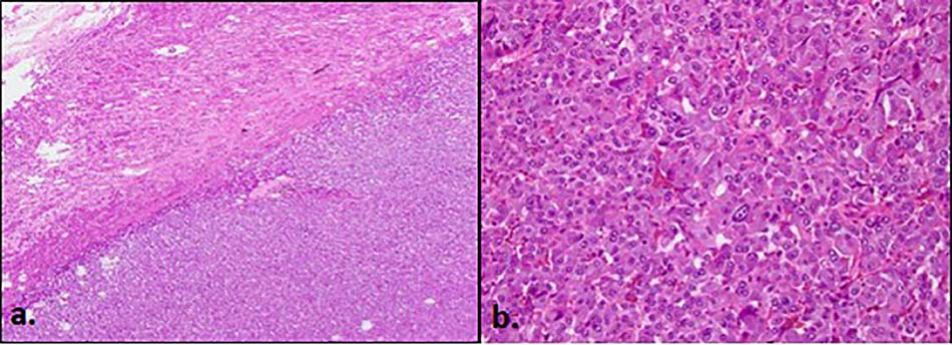
(**a**) ×2 magnification showing pheochromocytoma arising from medulla in relation to surrounding cortex and (**b**) ×20 magnification showing tumour cells arranged in classical zellballen patterns/nests with surrounding rich, large bore blood vessels. The nuclei of the cell show ‘salt and pepper’ chromatin. Not shown—ancillary studies including positive synaptophysin stain (neuroendocrine marker), positive S100 stain (sustenacular cells), retained SDHA and SDHB (succinate dehydrogenase subunit A and B), and low Ki-67 proliferation index (1%).

Endocrinology was consulted: biochemistry showed an elevated serum cortisol on dexamethasone suppression test (>1650 nmol/l; normal range < 50 nmol/l), and elevated early morning cortisol (>1649 nmol/l), adrenocorticotrophic hormone (ACTH; 15.7H pmol/l), renin (100 mIU/l) and aldosterone (835 pmol/l). Plasma metanephrines (normetadrenaline 20 000 pmol/l, normal range 130–1600; Metadrenaline 36 000 pmol/l, normal range 30–540) and 3 Methoxy Tyramine (682 pmol/l, normal range < 120) were also significantly elevated, suspicious for a secretory pheochromocytoma. She was commenced on alpha and beta blockade therapy (prazocin 2.5 mg/metoprolol 12.5 mg, once daily) and the upper gastrointestinal surgical team was consulted for elective resection after successful uptitration of her alpha blocker by cardiology/endocrinology teams. She was consented and proceeded for elective resection of her adrenal mass in the following 6 weeks when recovered, with pre-operative review in the high-risk anaesthetics clinic and planned post-operative transfer to ICU. Histology was positive for an organ-confined pheochromocytoma with no vascular/capsular invasion (Figs 2 and 3), consistent with T1NX on 8th Edition American Joint Committee on Cancer (AJCC) parameters [9]. She recovered well post-operatively under the care of the cardiology/endocrinology teams and was discharged one week later. Repeated echocardiogram at follow-up demonstrated normal left ventricular systolic function at 3 months.

## DISCUSSION

As secretory adrenal tumours, pheochromocytomas represent important, potentially reversible causes of TS. However, robust data on patients with TS secondary to pheochromocytoma is lacking. Larger datasets, such as the International Takotsubo Registry, (consisting of 9 countries and 1750 patients), excludes those with pheochromocytoma [3]. Although there have been over 80 published case reports of pheochromocytoma-induced TS (PTS; [7]), this is likely underreported, with a number erroneously reported as pheochromocytoma-induced angina or myocardial infarction, rather than TS per se [10]. We highlight three keyways PTS differs from TS—tendency to recurrence, symptom profile and complications. These differences are reflected in this case, and we hope may represent both diagnostic clues and considerations for other clinicians.

Both meta-analysis and case reports support the idea of recurrence. In a recent meta-analysis of 156 cases comparing catecholamine-induced (including pheochromocytoma amongst other secretory tumours and external administration) and non-catecholamine induced TS, Y-Hassan [8] and Falhammar found the catecholamine-induced TS group exclusively demonstrated recurrence rate (16.8%; 18/107; [8]). Preliminary data from the German Italian Spanish Takotsubo (GEIST) registry of 839 patients also found greater recurrence (17.7% vs 3.26%) [3, 7]. In cases of recurrence, normalization of left ventricular function is found on repeat echocardiogram (seen in our patient). From the literature, time between presentations is highly variable, ranging from 1 week to 6 years [10–12], with a number of presentations between 2 and 3 [10–12].

Catecholamine surges and sympathetic nervous system hyper-activation represents core pathophysiology of TS. Although catecholamine surge is associated with pheochromocytomas, the pathophysiology is not completely elucidated and this may be reflected in variability in presentations between PTS and TS. The symptomatology of adrenal tumours in general is broad and non-specific, and may be affected by sex. Regardless of histological subtype, both TS [4] and adrenal tumours [13] are more common in postmenopausal women, with advancing age and estrogen-deficiency risk factors for vasomotor changes and endothelial dysfunction. In addition, although signs and symptoms of pheochromocytoma are variable and non-specific, females have also been suggested to present with significantly more symptoms irrespective of biological phenotype and tumour presentation [14]. Compared with other causes of TS, PTS are less likely to be associated with chest pain (42.25 vs 75.9%), and present with labile blood pressure in nearly half (47.5%) of cases [7]. In our case, hypertension did not occur. Despite its well-known association, it is important to keep in mind that hypertension is an unreliable symptom of pheochromocytoma in general—occurring in only 59% of cases [15]—adding to diagnostic complexity.

The severity of complications in PTS compared to TS should also keep PTS an important differential. Catecholamine-induced TS are more likely to be associated with younger age, the apical sparing ballooning pattern on echocardiogram, more severe disease with lower LVEF (27.7 vs 41.1%), greater heart rate, greater complications (68.2 vs 21.8%), more cardiogenic shock (37.7 vs 9.9%) and greater use of inotropes (32.7 vs 12.2%; [3, 7]). Of published case reports, only three cases presented with cardiogenic shock, with a larger number (34.6%; 27/78) complicated by subsequent cardiogenic shock [7]. The GEIST registry found right ventricular involvement (as seen in our patient) in TS to be rare (~11%), and significantly associated with cardiogenic shock and all-cause mortality [16]. Symptom severity and greater complication rates may be attributable to the episodic and extreme nature of catecholamine surges in pheochromocytoma, which would impair coronary vasomotor function [17], and over a prolonged period, may influence density of myocardial B1–B2-adrenoreceptors, 1^2^ leading to further dysfunction.

The atypical nature of PTS may lead to diagnostic delays, as in this case, which is concerning given the increased rate of complications in this subgroup. As Shams et al. [10] note, early diagnosis may avoid further damage caused by excessive use of inotropes, as well as use of beta blockers without adequate, prior alpha blockade. Hence, we suggest secretory adrenal tumours should be considered as a differential diagnosis for Takotsubo’s Syndrome—particularly, a subsequent presentation of sympathetic hyperactivation, and presentations in the post-menopausal female, should give clinicians a low threshold for screening.

## FUNDING

The authors received no financial support for the research, authorship, and/or publication of this article.

## CONFLICTS OF INTEREST

The authors have no conflicts of interest to disclose.

## Supplementary Material

Abstract_omac066

ACT_Health_Low_Risk_Research_Consent_Form_Template_omac066

## References

[1] Dote K , Sato H, Tateishi H, Uchida T, Ishihara M. Myocardial stunning due to simultaneous multivessel coronary spasms: a review of 5 cases. J Cardiol 1991;21:203–14. 1841907

[2] Shams Y . Acute cardiac sympathetic disruption in the pathogenesis of the takotsubo syndrome: a systematic review of the literature to date. Cardiovasc Revasc Med 2014;15:35–42. 24140050 10.1016/j.carrev.2013.09.008

[3] Templin C , Ghadri JR, Diekmann J, Napp LC, Bataiosu DR, Jaguszewski M et al. Clinical features and outcomes of takotsubo (stress) cardiomyopathy. N Engl J Med 2015;373:929–38. 26332547 10.1056/NEJMoa1406761

[4] Pelliccia F , Kaski JC, Crea F, Camici PG. Pathophysiology of Takotsubo syndrome. Circulation 2017;135:2426–41. 28606950 10.1161/CIRCULATIONAHA.116.027121

[5] Gianni M , Dentali F, Grandi AM, Sumner G, Hiralal R, Lonn E. Apical ballooning syndrome or takotsubo cardiomyopathy: a systematic review. Eur Heart J 2006;27:1523–9. 16720686 10.1093/eurheartj/ehl032

[6] Nef HM , Möllmann H, Kostin S, Troidl C, Voss S, Weber M et al. Tako-Tsubo cardiomyopathy: intraindividual structural analysis in the acute phase and after functional recovery. Eur Heart J 2007;28:2456–64. 17395683 10.1093/eurheartj/ehl570

[7] Shams Y . Clinical features and outcome of pheochromocytoma-induced takotsubo syndrome: analysis of 80 published cases. Am J Cardiol 2016;117:1836–44. 27103159 10.1016/j.amjcard.2016.03.019

[8] Y-Hassan S , Falhammar H. Clinical features, complications, and outcomes of exogenous and endogenous catecholamine-triggered Takotsubo syndrome: a systematic review and meta-analysis of 156 published cases. Clin Cardiol 2020;43:459–67. 32125009 10.1002/clc.23352 PMC7244299

[9] Amin MB. ES, Greene F, Byrd DR, Brookland RK, Washington MK, Gershenwald JE, Compton CC, Hess KR, et al. AJCC Cancer Staging Manual, 8th edn. New York: Springer, 2017.

[10] Shams Y . Recurrent takotsubo syndrome triggered by undiagnosed pheochromocytoma. Int J Cardiol 2015;187:369–71. 25841128 10.1016/j.ijcard.2015.03.220

[11] Rossi AP , Bing-You RG, Thomas LR. Recurrent takotsubo cardiomyopathy associated with pheochromocytoma. Endocr Pract 2009;15:560–2. 19491077 10.4158/EP09005.CRR1

[12] Paur H , Wright PT, Sikkel MB, Tranter MH, Mansfield C, O'gara P et al. High levels of circulating epinephrine trigger apical cardiodepression in a β2-adrenergic receptor/Gi–dependent manner: a new model of Takotsubo cardiomyopathy. Circulation 2012;126:697–706. 22732314 10.1161/CIRCULATIONAHA.112.111591 PMC4890655

[13] Audenet F , Méjean A, Chartier-Kastler E, Rouprêt M. Adrenal tumours are more predominant in females regardless of their histological subtype: a review. World J Urol 2013;31:1037–43. 23299088 10.1007/s00345-012-1011-1

[14] Lai EW , Perera SM, Havekes B, Timmers HJ, Brouwers FM, McElroy B et al. Gender-related differences in the clinical presentation of malignant and benign pheochromocytoma. Endocrine 2008;34:96–100. 18982461 10.1007/s12020-008-9108-4

[15] Baguet JP , Hammer L, Mazzuco TL, Chabre O, Mallion JM, Sturm N et al. Circumstances of discovery of phaeochromocytoma: a retrospective study of 41 consecutive patients. Eur J Endocrinol 2004;150:681–6. 15132724 10.1530/eje.0.1500681

[16] El-Battrawy I , Santoro F, Stiermaier T, Möller C, Guastafierro F, Novo G et al. Incidence and clinical impact of right ventricular involvement (biventricular ballooning) in Takotsubo syndrome: results from the GEIST Registry. Chest 2021;160:1433–41. 34052189 10.1016/j.chest.2021.04.072

[17] Sato K , Takahashi J, Amano K, Shimokawa H. A case of recurrent takotsubo-like cardiomyopathy associated with pheochromocytoma exhibiting different patterns of left ventricular wall motion abnormality and coronary vasospasm: a case report. Eur Heart J-Case Rep 2020;4:1–5. 10.1093/ehjcr/ytaa138 PMC7501897 32974441

