# Supplementary material for: The heart of the matter: secretory pheochromocytoma presenting as recurrent biventricular heart failure (Takotsubo cardiomyopathy)
Source: Oxf Med Case Reports. 2022 Jun 23;2022(6):omac066. doi: 10.1093/omcr/omac066 (PMC9235020; doi:10.1093/omcr/omac066)
Supplement: Abstract_omac066 [file abstract_omac066.docx]

**Abstract**

Takotsubo’s Syndrome is an acute, transient cardiomyopathy occurring secondary to physical or emotional stressors through catecholamine excess. Secretory pheochromocytomas have been previously implicated in cases of TS (PTS), however, often present atypically, are associated with reoccurrence, and have higher rates of complications. We describe the case of a 70-year-old-female who presented central chest pain, hypotension and ECG changes on a background of a 6-month prior episode of resolved Takotsubo’s with unknown cause. After progressing to cardiogenic shock with biventricular failure, CT Coronary Aortogram revealed an incidental adrenal mass, later proven to be a secretory pheochromocytoma on biochemistry and subsequent histology. PTS has been associated with recurrence and rarely presents as cardiogenic shock. This case highlights the complexity of TS presentations and complications and the diagnostic delays that may occur in PTS.

**Keywords:** Adrenal, pheochromocytoma, heart failure, takotsubo
