# Supplementary material for: The heart of the matter: secretory pheochromocytoma presenting as recurrent biventricular heart failure (Takotsubo cardiomyopathy)
Source: Oxf Med Case Reports. 2022 Jun 23;2022(6):omac066. doi: 10.1093/omcr/omac066 (PMC9235020; doi:10.1093/omcr/omac066)
Supplement: ACT_Health_Low_Risk_Research_Consent_Form_Template_omac066 [file act_health_low_risk_research_consent_form_template_omac066.docx]

**Patient Consent Form**

**Consent Form for Participation in a Research Project.**

I, _______________________________________ *(name of participant)*

of _________________________________________________________ *(address)*

have been asked to consent to participation in a case report undertaken for research purposes:

In relation to this study I have been informed of the following points:

- - 1. The aim of the study is to **for teaching purposes and to improve awareness of the condition**
    2. That this information will be published in a professional journal but may be presented at scientific meetings, teaching, or for other educational purposes
    3. That the results of this study may or may not be of direct benefit to me
    4. That the report will include deidentified health information including:
       - Pictures
       - Medical history
       - Diagnoses
       - Treatment information
    5. Should I have any problems or queries about the way in which the study was conducted, and I do not feel comfortable contacting the research staff, I am aware that I may contact the ACT Health Human Research Ethics Committee Secretariat, Canberra Hospital, Yamba Drive, Garran ACT 2605 (ph: 6174 7968)
    6. I can refuse to take part in this project or withdraw from it at any time without giving a reason
    7. I understand that while the results of the research will be made accessible my involvement and my identity will not be revealed.

After considering all these points, I accept the invitation to participate in this study.

**Name:** (please print) **__________________________Date: ______________**

**Signature** (Participant) **________________________**

**Investigator:** (please print) **________________________Date: ______________**

**Signature** (Investigator) **_______________________**
